# The effect of isokinetic hip muscle strength on normal medial longitudinal arch feet and *pes planus*

**DOI:** 10.25122/jml-2021-0319

**Published:** 2022-09

**Authors:** Fayez Alahmri, Saad Alsaadi, Mohammad Ahsan, Sulaiman Almousa

**Affiliations:** 1 Department of Medical Rehabilitation, Ministry of Health, Riyadh, Saudi Arabia; 2 Department of Physical Therapy, College of Applied Medical Sciences, Imam Abdulrahman Bin Faisal University, Dammam, Saudi Arabia; 3 Orthopedic Department, King Fahd Hospital of the University, College of Medicine, Imam Abdulrahman Bin Faisal University, Dammam, Saudi Arabia

**Keywords:** flat foot, *pes planus*, foot pronation, navicular test

## Abstract

*Pes planus* is a common foot and ankle physiologic deformity. The normal medial longitudinal arch is depressed or flattened due to a lack of strength in associated muscles, ligaments, and tendons. This study aimed to investigate how isokinetic hip muscular strength affected normal medial longitudinal arch feet and pea planus. Forty adult subjects participated in this study: 20 with pea planus and 20 with normal medial longitudinal arched feet. Both groups were similar in age (p=.074), weight (p=.324), height (p=.211), and BMI (p=.541). The navicular drop test determined the differences in navicular height. An isokinetic dynamometer was used to determine hip muscular strength (peak torque and total work) during hip flexion, extension, abduction, and adduction at speeds of 90°/s and 180°/s. A Kruskal-Wallis test was computed to determine the comparison between the normal medial longitudinal arch and pea planus. Subjects with normal medial longitudinal arch had more muscle strength than *pes planus*. Hip muscle strength did not show any significant difference between both groups. The abductor and adductor group muscles' total work were higher in subjects with *pes planus*. This study showed that normal medial longitudinal arched foot subjects have higher muscle strength than *pes planus*. However, the hip abductors were significantly lower in *pes planus* after measuring the total work, suggesting that individuals with *pes planus* are easily fatigued, possibly due to the overuse of the muscles that compensate for any changes in lower limb alignment.

## INTRODUCTION

*Pes planus* or foot pronation is a chronic disorder characterized by the absence of the medial longitudinal arch completely or partially and increased rearfoot eversion [1]. *Pes planus* involves plantar flexion and adduction of talus [2]. Internal rotation of the lower limb is generally associated with talus adduction [3, 4]. Forward motion is the most important biomechanical function of bipedal movement. Balance, shock absorption, and bodyweight support must all be maintained [5]. The *pes planus* cannot distribute the body's load due to the arch's increased flexibility, resulting in biomechanical changes [6]. Several musculoskeletal issues are caused by these changes, including knee injuries, Achilles tendinosis, lower back pain, and stress fracture [7]. It is possibly considered that *pes planus* is associated with internal rotation supination related to external rotation [8]. Understanding how motion is transferred from the distal to the proximal ends of the lower limbs and the proximal to the distal ends is critical for a person with *pes planus* [9]. Proximal injuries are likely linked to abnormal foot structures, while distal injuries may result in abnormal hip function. It has been found that *pes planus* is responsible for pelvic malalignment.

Previous studies showed that *pes planus* causes hip and pelvic misalignment and increased medial hip rotation in unilateral standing [10, 11]. Another study used a wedged sandal to simulate unilateral *pes planus* and looked at how it affected pelvic and lower extremity biomechanics during walking. They observed that *pes planus* caused enhancement in knee and hip moment and enhancement in internal rotation of the lower extremity [12]. However, as previously stated, the studies had some limitations. This study utilized a platform to replicate *pes planus* and recruited healthy subjects with normal medial longitudinal arch feet. However, different muscle activation patterns [13] and various levels of muscle strength [14] have been seen in subjects with *pes planus*, which may affect joint angles and kinematics. *Pes planus* may influence muscle strength given that *pes planus* is less rigid and produces less torque than a foot with a normal medial longitudinal arch [15]. It has been recommended that individuals with *pes planus* require additional muscular support during gait [16]. Therefore, if lower limb muscles are weak during gait, others may generate additional muscular power to compensate for the weak muscles [16, 17]. The impact of *pes planus* on hip muscle strength is currently unknown.

The current study explored the effect of hip muscle strength on *pes planus* (pronated foot) and normal medial longitudinal arch feet. To treat the whole lower extremity joint chain, it is critical to understand the impact of *pes planus* on the proximal joints. The current study could assist researchers and physical therapists in better understanding how the *pes planus* affects proximal joint function and mobility. As a result, the evaluation would be improved, conservative therapy will be used, and any future complications from *pes planus* will be avoided.

## MATERIAL AND METHODS

The study was based on an experimental design. The study occurred at Imam Abdulrahman Bin Faisal University's biomechanics lab in Dammam, Saudi Arabia. The study included forty adult males who volunteered to take part. The experimental group comprised 20 *pes planus*, and the control group comprised 20 individuals with normal medial longitudinal arched feet; both groups were similar in age, weight, height, and body mass index (BMI). The criteria for inclusion and exclusion were chosen based on their potential to affect muscle strength and alter the movement patterns of the lower limbs. The experimental group comprised subjects whose navicular drop (ND) test revealed a fall in arch height of more than 10 mm and whose rearfoot (calcaneal) angle was greater than 5 degrees. If a subject had any foot deformities, systemic or neurological illnesses, a history of foot or ankle surgery, or a recent fracture, they were ruled out from the study. The following criteria were used to choose the control group: ND test arch height change of less than 10 mm in a typical medial longitudinal arch foot and no lower limb diseases or fractures. Subjects who volunteered to participate in the study were given a detailed written and verbal explanation of the study's procedures and protocols.

The outcome measures included peak torque, a measure of a muscle's force capabilities determined from an isokinetic dynamometer device and recorded as the greatest muscular force created during the repetitions. Total work, or the amount of muscular work, is an indicator of the capability of the muscles to generate force throughout a range of motion. The isokinetic dynamometer system measured it. The researcher measured the subject's body weight and height when he arrived at the lab. The dominant leg of each subject was determined by asking him which leg he could use to kick a ball harder. The navicular drop (ND) test assesses the medial longitudinal arch height by measuring the differences in the navicular height between a non-weight-bearing (open kinetic) chain and a weight-bearing (closed kinetic) chain. The ND test is reliable and valid [18]. While seated, each subject placed one foot on a stable surface with the knee flexed at 90 degrees and the ankle joint in a neutral position; then, the tubercle of the navicular bone was marked with a pen, and an index card was placed on the inner side of the hindfoot perpendicular to the ground and adjacent to the navicular bone. The subject was requested to stand without moving his feet and distribute his weight evenly on both feet while the tubercle of the navicular bone was marked on the card, and the navicular was measured once again. Finally, a ruler measured the distance between the navicular bone while sitting and standing. ND under 10 mm was considered normal, and ND over 10 mm was considered a *pes planus* [18]. The rearfoot angle is a clinical measurement used in many studies [3, 19, 20] to assess calcaneal eversion or heel valgus and determine the amount of *pes planus*. Kanatli et al. [21] indicated that the rearfoot angle and the foot medial longitudinal arch height must be examined separately in *pes planus* assessments. The rearfoot angle was measured with the subject lying prone, and the foot and ankle extended 10 cm off the bed. The subject stood on both feet after drawing a longitudinal line with a pen along the rear side of the lower third of the leg. A goniometer was used to measure the angle between the calcaneus and the bottom portion of the tibia.

### Hip muscle strength measurement

Muscle strength was measured by the isokinetic dynamometer system (Biodex Medical Systems, Shirley, New York). The system controls the speed of a movement, allowing the subject to accelerate but not higher than the maximum speed selected for the protocol (accommodating resistance). The isokinetic Biodex system has moderate to high reliability in determining hip muscle strength [22]. Hip muscle strength was measured at 90 and 180 degrees per second at two different speeds. Each subject was given a detailed explanation of the procedure and test protocol. Each subject was allowed to warm up by cycling for 5 minutes to prepare the muscles for testing and prevent injuries. The calibration of the dynamometer was conducted according to the manufacturer's guidelines. Subject age, height, and weight were recorded. The dynamometer height, angle, rotation, and position were also adjusted. Stabilization straps were applied to stabilize the subject and isolate the muscles. The movement start and endpoints were selected according to the anatomical range of motion. The isokinetic dynamometer calculated the weight of the examined limb in a relaxed position. Before testing, each subject performed practice repetitions at the same speed to become familiar with the testing protocol. The testing protocol included concentric movements at 90°/s and 180°/s, with one minute rest period between each set. A rest of five minutes was also provided to test different muscle groups. Visual feedback from the Biodex system was provided on a computer monitor. In addition, the examiner provided verbal encouragement.

### Hip abduction/adduction test

The subject was lying on his side with the hip to be tested on top, facing away from the dynamometer, and his opposite limb was flexed at the knee. The attachment length was adjusted to place the pad superiorly to the popliteal fossa. The knee was fully extended and fastened at the femur level to prevent hip rotation. The trunk and pelvis were fastened to the chair. The starting position of the hip joint was at full adduction. The testing protocol included concentric movements at 90 and 180 degrees. The subject was asked to move his leg upward and downward by exerting his maximum strength for five repetitions when the testing started. Subjects were verbally encouraged and given visual feedback from the Biodex system.

### Hip flexion/extension test

The subject was seated in a supine position with the chair back flat. The tested hip was at 0 degrees of flexion with 90° of knee flexion. The attachment length of the hip was adjusted so that the thigh support was just superior to the popliteal fossa. A strap secured the non-tested thigh to the chair at 0-degree flexion of the hip. The trunk and pelvis were strapped as well. The testing protocol included concentric movements at 90 and 180°. Then, when testing started, the subjects were asked to flex and extend their hips by exerting their maximum strength for five repetitions.

### Statistical analysis

For statistical analysis, data were computed with the help of SPSS software version 23 for windows. The normality of data was checked by the Shapiro-Wilk test and found that data were not normally distributed. Thus, a non-parametric test (Kruskal-Wallis) was used to determine the difference between two groups. A Kruskal-Wallis test was employed if the two groups were similar in terms of age, height, weight, and BMI. The arch height and rearfoot angle differences were determined using the same test. Similarly, to evaluate and examine the differences between the two groups, a Kruskal-Wallis test was used concerning hip muscle strength determined by total muscular work and peak torque during hip flexion, extension, adduction, and abduction.

## RESULTS

### Subjects' characteristics

This study included forty adult males: 20 with *pes planus* and 20 with normal medial longitudinal arched feet. The characteristics of the subjects are shown in Table 1. No statistically significant differences existed for any of the variables, which indicates the groups had similar general characteristics. However, there were significant differences in arch height (p=0.001) and rearfoot angle (p=0.001).

**Table 1:** Descriptive statistics of subject's anthropometric characteristics and rare foot angle.

Characteristics	Pes Planus (n=20) Means±SD	Control (n=20) Means±SD	Sig.
Age (years)	20.64±5.7	21.64±3.4	0.744
Height (cm)	172.46±4.9	171.61±6	0.211
Weight (kg)	68.47±10.82	69.72±11.24	0.324
BMI (kg/m^2^)	21.4±4.7	24.7±5.2	0.541
Arch height ND (mm.)	12.6±2.26	7.5±.94	0.001
Rear-foot angle (degrees)	8.5±2.3	3.62±0.8	0.001

There were no significant differences between the *pes planus* group and the normal medial longitudinal arch feet in the peak torque and total work of the hip flexors, extensors, abductors, and adductors, as shown in Tables 2–5. However, there were significant differences in hip abduction total work but no significant differences in hip flexors, extensors, or adductors total work. Peak hip flexor torque and total work at 90 and 180 degrees per second.

**Table 2:** Comparison of hip muscle strength between pes planus and normal medial longitudinal arch feet during flexion.

	Speed	Groups	Grand Median	Independent-Samples Test Statistic	Asymptotic Sig. (2-sided test)
**Peak Torque**	90	*Pes Planus*	40.00	.752	.685
		Control			
	180	*Pes Planus*	52.00	.752	.766
		Control			
**Total Work**	90	*Pes Planus*	70.95	.752	.675
		Control			
	180	*Pes Planus*	57.20	.752	.735
		Control			

Control – Normal medial longitudinal arch feet; Sig. – p-value, with a significance level of P<0.05.

**Table 3:** Comparison of hip muscle strength between pes planus and normal medial longitudinal arch feet during extension.

	Speed	Groups	Grand Median	Independent-Samples Test Statistic	Asymptotic Sig. (2-sided test)
**Peak Torque**	90	*Pes Planus*	61.20	.752	.262
		Control			
	180	*Pes Planus*	52.95	.343	.387
		Control			
**Total Work**	90	*Pes Planus*	67.00	.343	.190
		Control			
	180	*Pes Planus*	46.95	.752	.417
		Control			

Control – Normal medial longitudinal arch feet; Sig. – p-value, with a significance level of P<0.05.

**Table 4:** Comparison of hip muscle strength between pes planus and normal medial longitudinal arch feet during abduction.

	Speed	Groups	Grand Median	Independent-Samples Test Statistic	Asymptotic Sig. (2-sided test)
**Peak Torque**	90	*Pes Planus*	51.60	.752	.914
		Control			
	180	*Pes Planus*	44.65	.752	.490
		Control			
**Total Work**	90	*Pes Planus*	26.00	.150	.036
		Control			
	180	*Pes Planus*	16.15	.752	.588
		Control			

Control – Normal medial longitudinal arch feet; Sig. – p-value, with a significance level of P<0.05.

**Table 5:** Comparison of hip muscle strength between pes planus and normal medial longitudinal arch feet during adduction.

	Speed	Groups	Grand Median	Independent-Samples Test Statistic	Asymptotic Sig. (2-sided test)
**Peak Torque**	90	*Pes Planus*	31.65	.752	.337
		Control			
	180	*Pes Planus*	26.55	.752	.144
		Control			
**Total Work**	90	*Pes Planus*	12.25	.343	.044
		Control			
	180	*Pes Planus*	8.25	.752	.818
		Control			

Control – Normal medial longitudinal arch feet; Sig. – p-value, with a significance level of P<0.05.

Table 3 showed no significant differences between the *pes planus* and normal medial longitudinal arch feet groups during extension in peak torque and total muscular work and hip extensor. Hip extensor peak torque and total work at speeds of 90 and 180 deg./s.

Table 4 showed no significant differences between the *pes planus* and normal medial longitudinal arch feet groups during abduction in peak torque and total muscular work of the hip abductor. Hip adductor peak torque and total work at speeds of 90 and 180 deg./s. At the same time, only total muscular work of the hip abductor was significant (p=.036) at a speed of 90 deg./sec.

Table 5 showed no significant differences between the *pes planus* and normal medial longitudinal arch feet groups during adduction in peak torque and total muscular work of the hip adductor. Hip adductor peak torque and total work at speeds of 90 and 180 deg./s. At the same time, only total muscular work of the hip adductor was significant (p=.044) at a speed of 90 deg./sec.

## DISCUSSION

The study compared isokinetic hip muscle strength. The findings revealed no significant differences in hip muscle strength between *pes planus* and normal medial longitudinal arched feet. However, the total work of the hip abductor muscles was substantially lower in the *pes planus* group. Only the total muscular work showed significant differences between normal medial longitudinal arch feet and *pes planus* for abduction and adduction at 90 deg./sec. Our findings agree with previous reports showing no significant differences in muscle strength between *pes planus* and normal arched foot. For example, according to Lizis et al. [23], foot arch height was not significantly associated with muscle strength. Their study regarding the relationship between lower limb muscle strength and foot arch height concluded that flexible flat feet should be considered within the normal range of a strong and stable foot and rarely causes disability. A study investigates the impact of arch height on knee and ankle muscular strength. There was no significant difference in ankle muscle strength or *pes planus* grades in adult females aged 18–24 years. The absence of substantial differences between the groups may be attributable to our young and active patients [24]. The influence of *pes planus* on ankle muscle peak torque was investigated by Karatsolis et al. who revealed insignificant differences for peak torque measurements between *pes planus* and normal medial longitudinal arched foot groups [25]. Zhao et al. investigated how arch heights affected muscular strength in 67 subjects. They tested the strength of the ankle dorsiflexor and plantar flexor muscles as well as the ankle invertor and evertor muscle groups at 30°/s and 120°/s. They discovered a link between high arch level and ankle muscular strength, with persons with low arch levels scoring higher on muscle strength tests [26]. Aydog et al. [27] examined the relationship between ankle joint muscle strength and foot posture, and their result showed no relationship between ankle joint muscle strength and arch height. Those who have *pes planus* have developed to adopt the structural abnormality. People with *pes planus* had higher and varied muscle activations and relied on additional muscular support during gait than people without *pes planus* [16]. Fayez et al. reported no significant differences between asymptomatic pronation foot and non-pronated foot, with the kinematic analysis being done for the hip joint from three different directions [28]. Ransimala et al. investigated the relationship between *pes planus* and hip abductor muscle strength among male and female undergraduate students. While comparing the hip abductor muscle strength, they found that the average hip abductor muscle strength in the right leg was 76.17, whereas the average hip abductor muscle strength in the left leg was 73.12. The left and right abductor muscle strength in males and females had a significant value of 0.00. They discovered a statistical significant difference in hip abductor muscle strength between females and males when looking at both flat-footed and non-flat-footed student groups [29].

The present study showed insignificant differences between *pes planus* and normal medial longitudinal arch feet for hip muscle strength as peak torque and total work done by the muscles during isokinetic contraction. The subjects with normal medial longitudinal arch had more flexor, extensor, abductor, and adductor muscle strength (peak torque) than those with *pes planus* at speeds of 90 and 180 deg./s.

The total muscle work means that the score is higher in flexor and extensor group muscles for subjects with pes planes at speeds of 90 and 180 deg./s. Only the total muscular work showed significant differences between normal medial longitudinal arch feet and *pes planus* for abduction and adduction at 90 deg./sec. The total work of the abductor and adductor group muscles was higher in subjects with *pes planus* than in subjects with normal medial longitudinal arch feet. The hip abductors and adductors of the subjects with *pes planus* generated less muscular work than those of the normal medial longitudinal arch feet, which may be due to overuse of the muscle to compensate for abnormalities and may indicate that individuals with the pronated foot are easily fatigued.

The current study has some limitations. First, all our subjects were males, making it difficult to generalize the findings for all populations. The second limitation was that the subjects in this study were all of young age, mean 21 years. The third limitation was the study design, which could not see the long-term effect of deformity on muscle strength. In addition, a smaller number of subjects were selected to conduct this study, especially when analyzing groups. Finally, as the subjects were from only the eastern region, the results can be biased and influenced by factors associated with this region.

## CONCLUSIONS

The current study analyzed the isokinetic muscle strength between *pes planus* and normal medial longitudinal arch feet in adults. We found that individuals with *pes planus* and those with normal medial longitudinal arch feet were similar in hip muscle strength at flexion, extension, abduction, and adduction.

## Personal thanks

We gratefully acknowledge the approval of the Dean and Head of the Department of Physical Therapy, College of Applied Medical Sciences, Imam Abdulrahman Bin Faisal University, Dammam, to perform this study. Aside from that, we want to thank all the subjects who participated in this study.
